# Cutaneous squamous-cell carcinoma of the head-neck area refractory to chemo-radiotherapy: benefit from anti-PD-1 immunotherapy

**DOI:** 10.1259/bjrcr.20200170

**Published:** 2021-02-04

**Authors:** Ioannis M. Koukourakis, Axiotis G. Giakzidis, Maria Kouroupi, Alexandra Giatromanolaki, Ioannis Abatzoglou, Antonios Karpouzis, Michael I. Koukourakis

**Affiliations:** 1 Department of Radiotherapy – Oncology, Medical School, Democritus University of Thrace, Alexandroupolis, Greece; 2 Department of Pathology, Medical School, Democritus University of Thrace, Alexandroupolis, Greece; 3 Department of Dermatology, Medical School, Democritus University of Thrace, Alexandroupolis, Greece

## Abstract

**Objective:**

Radiotherapy provides excellent results in locally advanced cutaneous squamous-cell carcinoma of the head and neck area (cSCC-HN), with a 2-year local progression-free interval obtained for about 80% of patients. Overexpression of immune checkpoint co-inhibitory molecules, like PD-L1 (programmed death ligand 1), by cancer cells may define local immunosuppression, tumour escape from immune surveillance and reduced radiotherapy efficacy.

**Methods:**

A 65-year-old female, with a large exophytic cSCC-HN invading adjacent soft tissues, was treated with hypofractionated accelerated chemo-radiotherapy. The patient received four bi-weekly cycles of chemotherapy concurrently with eight fractions of 5.5 Gy (two fractions per week). Two months after the end of chemo-radiotherapy, the tumour was stable in dimensions, without any signs of symptomatic relief. The patient was, after that, treated with anti-PD-1 immunotherapy (nivolumab). The tumour gradually regressed, reaching partial response after four cycles and complete response after 16 cycles of nivolumab. No side-effects related to immunotherapy were recorded. The patient is alive and without evidence of disease 28 months after radiotherapy.

**Conclusions:**

Treatment of patients with chemo- and radio-resistant cSCC-HN with immunotherapy may optimize the efficacy of radiotherapy by stimulating immunological tumour rejection mechanisms. cSCC-HN patients who fail to respond to chemo-radiotherapy completely are expected to benefit the most from immunotherapy because of the radio-vaccination effect expected from the preceded radiotherapy.

## Introduction

Radiotherapy is the treatment of choice for locally advanced cutaneous squamous-cell carcinoma of the head and neck area (cSCC-HN). Although a 2-year local progression-free interval is reached for about 80% of patients, this is reduced down to 47% in immunocompromized patients, implying an essential role of the immune system in the efficacy of radiotherapy^1^. In immunocompetent patients, the escape of tumours from immune surveillance may occur for several reasons, including the overexpression of immune checkpoint co-inhibitory molecules (ICI-molecules) by cancer cells^2^.

PD-L1 (programmed death ligand 1) is a critical ICI-molecule that, once expressed by cancer cells, blocks the cytotoxic activity of T-cells that attack the tumour, by binding to the PD-1 receptor expressed on the cytotoxic T-cell surface.**^3^** Anti-PD-L1 and anti-PD-1 monoclonal antibodies have shown important anti-tumour activity and are used for the treatment of melanomas, renal cell carcinomas, lung, head-neck and bladder carcinomas, or even for any tumour that has high tumour mutational burden (TMB), high microsatellite instability (MSI-high) or mismatch-repair deficiency (dMMR)^4^.

The current case report provides strong evidence that fighting local immunosuppression through the available anti-PD-1/PD-L1 therapies may prove critical for the elimination of cSCC-HN refractory to radiotherapy.

## Case presentation

A 65-year-old female, with an exophytic, 8 cm in diameter (horizontal 8 cm, depth 5 cm) cSCC-HN, located to the left acromion-clavicular area, was referred to our department on April 2018. The tumor had recurred following two incomplete surgical resections, performed during the past year. Symptomatology included pain and discomfort in the movement of the shoulder. CT-scan revealed the mass that was infiltrating deep into the soft tissues. There was no evidence of nodal or distant metastatic disease.

## Treatment

**Figure 1.**
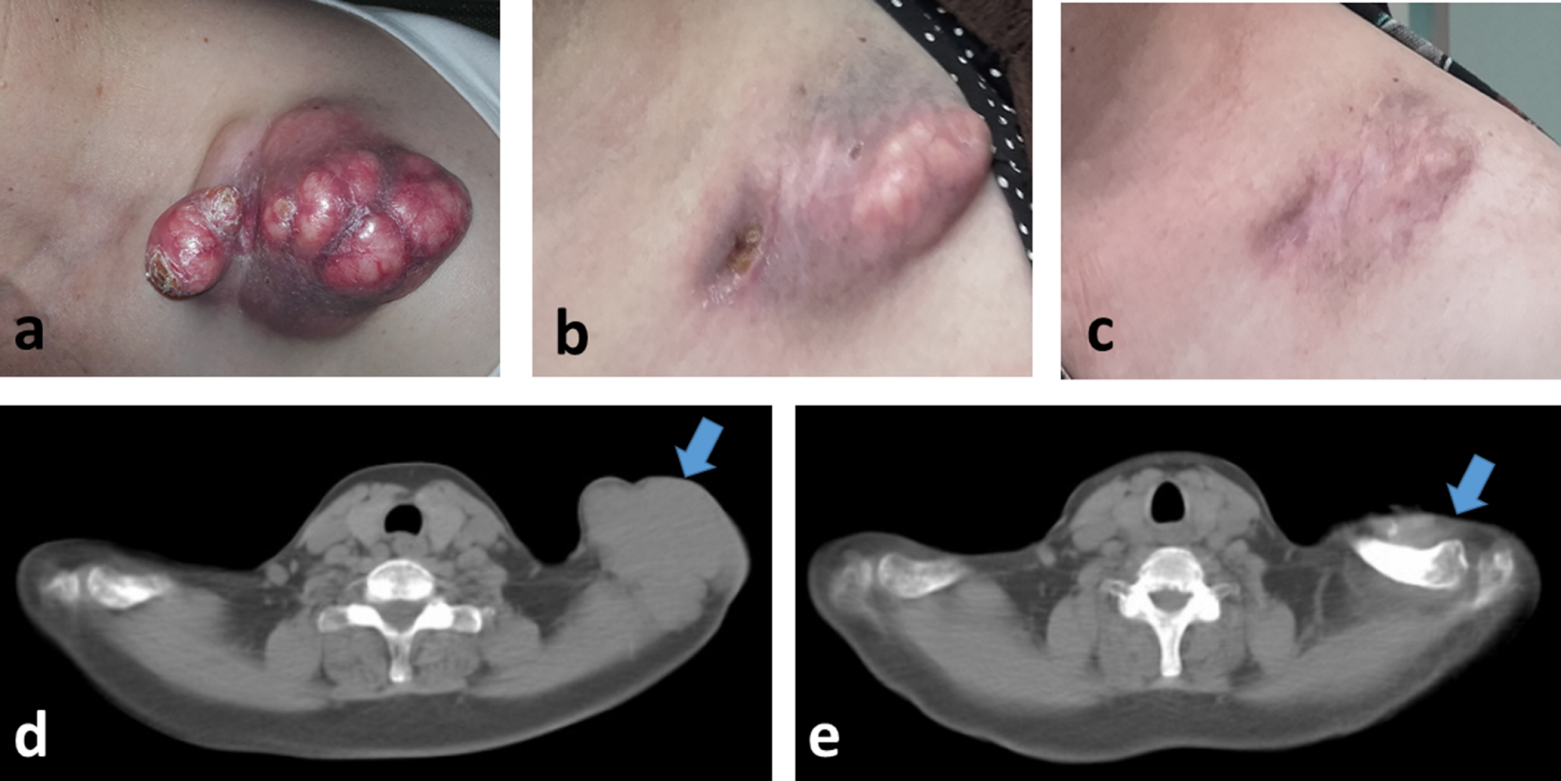
A cutaneous squamous-cell carcinoma of the acromion/supraclavicular area, two months after the end of chemo-radiotherapy (**a,d**), after four cycles (**b**) and 16 cycles (**c,e**) of nivolumab immunotherapy.

The patient was treated according to a protocol of hypofractionated accelerated cisplatin and/or cetuximab-based chemo-radiotherapy for locally advanced inoperable head-neck cancer, approved by the local Ethics and Scientific Committee (DS7/26-2-2004 and DS34/28-9-2006). Treatment of patients with cancer refractory or relapsing after chemo-radiation with anti-PD-1 therapy has been approved by the local Ethics and Scientific Committee (ES7/2019). Written informed consent was obtained from the patient. The patient received four bi-weekly cycles of chemotherapy with cisplatin (50 mg/m2), cetuximab (400 mg/m2) and 5-fluorouracil (600 mg/m2). These are among the drugs widely used for advanced cSqHNC.**^5^** This was delivered concurrently with an accelerated hypofractionated scheme of radiotherapy, delivering eight fractions of 5.5 Gy (two fractions per week) to the tumour.**^6^** The normalized total biological dose (EQD2; equivalent to a 2Gy-per-fraction regimen) delivered to the patient, calculated for normal tissue toxicity (α/β = 4 Gy), without time correction, was 66 Gy in 3.5 weeks. The EQD2 for skin cancer (α/β = 10 Gy^7^) is estimated to 56.8 Gy, delivered within 3.5 weeks, thus with an acceleration of 12 days. Assuming a minimum λ-value of 0.4 Gy^8^ , the EQD2 with time correction to the tumour is estimated at 61.6 Gy. Radiotherapy was performed with a combination of 6 MV X-rays (five fractions) and 15MeV electrons (three fractions), using a Linear Accelerator (Elekta, Synergy). This energy was adequate to cover the 5 cm depth dimensions of the tumour. Two months after the end of chemo-radiotherapy, the tumour was stable in dimensions, without any signs of pain relief (Figure 1a and d).

Thus, the disease was considered resistant to radiotherapy and cisplatin-based chemotherapy. It was, therefore, decided to further treat the patient with immunotherapy. As Cemiplimab has become only recently available in Greece for the treatment of squamous-cell skin cancer, the patient received nivolumab (240 mg every two weeks), a drug approved for advanced head-neck cancer. Nivolumab is an anti PD-1 monoclonal antibody approved for the treatment of advanced head-neck cancer, following a positive randomized trial (CheckMate-141) that confirmed higher, still low (13.3%), objective response rates compared to chemotherapy.**^9^** Although the administration of the antibody has been approved independently of the PD-L1 expression status of cancer cells, we performed immunohistochemistry to assess the expression of PD-L1 and genes involved in mismatch-repair deficiency (MLH1, PMS2, MSH2, and MSH6). The tumour showed minimal PD-L1 expression, concerning 1% of cancer cells, and extensive loss of expression of all four dMMR genes.

## Outcome and Follow-up

Two weeks after the first cycle of immunotherapy, the tumour showed signs of regression, and partial response was obtained after the fourth cycle of nivolumab (Figure 1b). Complete remission of the pain and restoration of the shoulder mobility was achieved gradually. Following 16 cycles of immunotherapy (8 months), the tumour has reached complete response (Figure 1c and e). No side-effects related to immunotherapy were recorded. The patient is alive and without evidence of disease 28 months after radiotherapy.

## Discussion

Although radiotherapy is highly effective in cSCC-HN, radio-resistant tumours are often encountered in clinical practice. This refractory group of patients is, most often, resistant to further chemotherapy. Patients die from progressive local and distant disease, with a poor quality of life that is compromised by the pain, the disfiguration or, even, the immobilization from muscle and bone infiltration by the tumour. The 20% rates of failure of radiotherapy to control cSCC-HN may be related to immunological pathways exploited by cancer cells to evade immune surveillance^1^ , aside to other reasons like intrinsic radio-resistance.

Anti-PD-1 therapy has shown important activity against squamous-cell carcinomas of the head-neck area, with reported response rates ranging from 13 to 18%^10^. These relatively low response rates are, nevertheless, higher than the 6–10% rates reported with various chemotherapy agents. Cemiplimab, an anti PD-1 MoAb, has been recently approved for the treatment of advanced squamous cell skin cancer. The response rates to cemiplimab are high, ranging between 40 and 50%, but the complete response rates were 0–7%.**^11^** Nevertheless, several previous reports confirm the activity of nivolumab against skin cancer^12–14^. As previous exposure to radiotherapy seems to increase the efficacy of immunotherapy, anti PD-1 therapy for irradiated cSCC-HN may be strongly effective. Although this has not been documented in HNC or cSCC-HN, the Keynote 001 Phase I study on the activity of pembrolizumab (anti PD-1 monoclonal antibody) in non-small cell lung cancer, suggested that patients who had been previously exposed to radiotherapy had better responses and prolonged survival^15^. Restoration of anti-PD-1 immunotherapy efficacy and documentation of abscopal effects, when adding radiation to immunotherapy, have also been reported, suggestive of a radio-vaccination effect for the benefit of patients^16^. A large number of experimental studies have elucidated the complex radio-vaccination pathways, including activation of the IFN type I pathway, enhanced antigen presentation by HLA-class-I molecules and cytokine release by irradiated cancer cells^17^. Of interest, large radiotherapy fractions, even higher than the ones used in the current case report, seem to be necessary to induce radio-vaccination^18^.

The herein reported case is a typical example of a radio- and chemo-resistant cSCC-HN. Although some tumour types, like melanomas, sarcomas or even, occasionally, squamous-cell carcinomas of the lung and head-neck area may exhibit a very slow regression that becomes measurable months after treatment completion, in our experience, radio-sensitive squamous skin carcinomas show a clear evidence of response within the first weeks after therapy. Generally, early detection of even small persistent disease two months after radiotherapy calls for increased awareness. For example, therapeutic interventions like surgery or stereotactic radiotherapy, are strongly recommended in persistent residual nasopharyngeal cancer, as it is most often followed by tumour progression.**^19^** Immunotherapy was a sound option to treat our patient, as the striking lack of tumour regression predicted for a local relapse with a lethal outcome. Cemiplimab was not available in 2018 in Greece, so our patient was offered nivolumab, a MoAb approved for the treatment of locally advanced head-neck cancer. The gradual regression of the tumour, till complete disappearance, support that some cSCC-HN are tumours where cancer cell-induced local immunosuppression is among the causes of chemo-radiotherapy failure.

Immunotherapy has undoubtedly an important role in the treatment of recurrent and metastatic squamous-cell head-neck cancer. An additional group of patients is the one who fails to respond to chemo-radiotherapy completely. For head-neck cancer or cSCC-HN, there are no randomized trials to reveal the role of post-radiotherapy immunotherapy for residual tumours. Experimental evidence supports that this is the group that would benefit the most from immunotherapy, as the persistent radio-vaccinated tumour is, eventually, more vulnerable to the immune system. This hypothesis has been substantiated in the ‘Pacific’ randomized trial in patients with non-small cell lung cancer, where post-chemo-radiotherapy anti-PD-L1 immunotherapy offered significant prolongation of survival.**^20^** Treatment of patients with chemo- and radio-resistant cSCC-HN with immunotherapy, immediately after documentation of residual gross disease, may provide a significant benefit, optimizing the efficacy of radiotherapy by stimulating immunological tumour rejection mechanisms. This hypothesis, supported by the current report, should be tested in large Phase II trials.

## Learning points

1.. Host immune response is an active component of the equilibrium achieved in post-radiotherapy residual disease.

2. Fighting local immunosuppression through the available anti-PD-1/PD-L1 therapies may prove critical for the elimination of cSCC-HN refractory to radiotherapy.
